# Post-Process Effects of Isothermal Annealing and Initially Applied Static Uniaxial Loading on the Ultimate Tensile Strength of Fused Filament Fabrication Parts

**DOI:** 10.3390/ma13020352

**Published:** 2020-01-12

**Authors:** Rhugdhrivya Rane, Akhilesh Kulkarni, Hardikkumar Prajapati, Robert Taylor, Ankur Jain, Victoria Chen

**Affiliations:** 1 Mechanical and Aerospace Engineering, University of Texas at Arlington, Arlington, TX 76019, USA; akhilesh.kulkarni@mavs.uta.edu (A.K.); hardikkumarmang.prajapati@mavs.uta.edu (H.P.); taylorrm@uta.edu (R.T.); jaina@uta.edu (A.J.); 2 Industrial, Manufacturing, & Systems Engineering, University of Texas at Arlington, Arlington, TX 76019, USA; vchen@uta.edu

**Keywords:** additive manufacturing, fused deposition modelling, interface healing, ultimate tensile strength, thermal annealing, uniaxial loading, post processing

## Abstract

Fused filament fabrication (FFF) is one of the most popular additive manufacturing (AM) techniques used to fabricate polymeric structures. However, these polymeric structures suffer from an inherent deficiency of weak inter-laminar tensile strength. Because of this weak inter-laminar strength, these parts fail prematurely and exhibit only a fraction of the mechanical properties of those fabricated using conventional means. In this paper, we study the effect of thermal annealing in the presence of an initially applied static uniaxial load on the ultimate tensile strength of parts fabricated using FFF. Tensile specimens or dogbones were fabricated from an acrylonitrile butadiene styrene (ABS) filament with a glass transition temperature (T_g_) of 105 °C; these specimens were then isothermally annealed, post manufacture, in a fixture across a given range of temperatures and static loads. Tensile testing was then performed on these specimens to gauge the effect of the thermal annealing and static loading on inter-laminar tensile strength by measuring the ultimate tensile strength of the specimens. A design of experiments (DOE) approach was followed to calculate the main and interaction effects of the two factors (temperature and static loading) on the ultimate tensile strength, and an analysis of variance was conducted. Cross-sectional images of the specimens were studied to observe the changes in the mesostructure of the specimens that led to the increase in inter-laminar strength of the parts. The results show that temperature plays a dominant role in increasing the ultimate tensile strength and an 89% increase in the average ultimate tensile strength was seen corresponding to an annealing temperature of 160 °C. A change in the mesostructure of the parts is seen, which is characterized by an increase in bond length and void coalescence. These results can be helpful in studying the structural strength of 3D printed parts, and thus could eventually guide the fabrication of components with strength comparable to those of conventional manufacturing techniques.

## 1. Introduction

**Figure 1:**
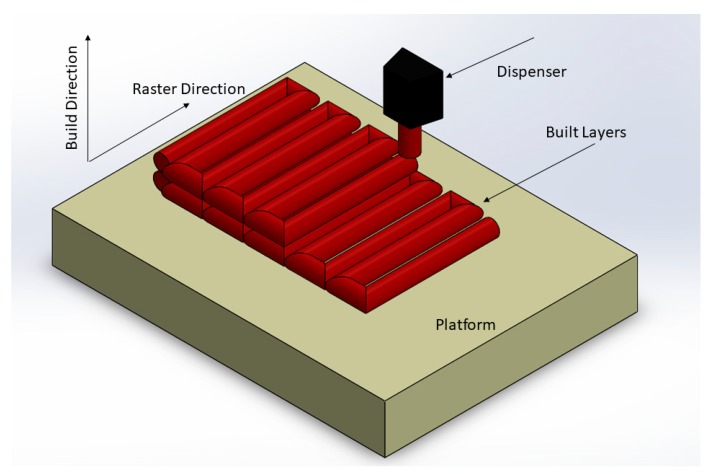
Schematic for filament rastering process in fused filament fabrication (FFF).

Manufacturing techniques have constantly been evolving with a greater emphasis on fabricating complex geometries with a high degree of dimensional accuracy. Additive manufacturing (AM) techniques are a set of disruptive techniques that have gained popularity owing to their ease of availability, comparatively cheaper feedstock, and capability to build part geometries that would otherwise be challenging to fabricate using traditional methods. Unlike traditional mold manufacturing techniques, which involve complex designing and are time-consuming, AM uses digital technology to build parts [1]. The AM processes have been segregated into seven categories by the ASTM F42 committee to standardize the terminology. These techniques differ from each other based on the type of feedstock used, the source of energy, and the method of material deposition. Fused filament fabrication (FFF) is one of the most commonly used AM technologies in terms of the number of parts produced, printers, and printer manufacturers worldwide [2]. FFF uses a Computer Aided Design (CAD) model to build an object in a layer by layer manner, as shown in Figure 1. Unlike many of the AM technologies, which have a source of energy that rasters over a powder bed, causing the powder to selectively fuse as a result of melting and then solidification to generate the final shape [3,4,5,6,7], in FFF, the extruder deposits the polymer melt onto a heated bed at a temperature above the glass transition temperature [3,4,8]. In FFF systems, a thermoplastic filament stock is forced through a heated liquefier with the help of a pinch roller. The print nozzle is moved in the x–y direction with the help of a gantry while depositing the material. The z-direction or out of plane movement is provided by either the bed moving downwards or the nozzle moving upwards. As the pinch rollers force feed the filament into the heated liquefier, the feedstock melts and the solid portion of filament acts as a piston pushing the melt through the nozzle [2]. FFF is highly popular owing to its comparatively low cost and simple feedstocks as compared with other AM technologies [9]. Owing to their popularity, the polymer-based AM manufacturing processes such as FFF have been extensively studied for a variety of applications [3,10]. However, the parts printed using FFF have reduced mechanical [11,12,13] and thermal properties [14] required in most end-use applications, and thus are not preferred in such engineering applications.

The inter-laminar bonding in FFF parts largely affects their mechanical properties. These parts are weaker in comparison with parts fabricated using traditional techniques like injection molding at the inter-laminar region and fail prematurely at such interfaces, thus dominating the overall strength of the parts. This failure at the inter-laminar region has been studied for a wide range of testing conditions including tension [15], compression [16,17], torsion [18], and fatigue [19,20,21]. These studies reinforce the understanding that this premature failure is influenced by the inter-laminar bonding. Attempts have been made to improve the inter-laminar bonding by employing a multitude of techniques, which include varying print parameters (i.e., printing temperature, raster speed, material deposition rate, air gap, layer thickness) [22,23,24], adding plasticizer to the filament [25], adding composite filaments [26], and microwave irradiation [27]. Some improvement in the mechanical performance is seen through these methods, but it is still insufficient when compared with the demands of engineering applications, and cannot match up with parts fabricated using injection molding.

Various print parameters like build orientation [28], air gap [18,29], and printing temperatures [30] affect the mechanical properties of FFF parts. However, the two main factors that result in subpar mechanical properties of FFF parts are porosity and imperfect weld-lines at the inter-laminar bonding interface [31]. Porosity is an inherent defect of FFF because it uses circular nozzles to deposit the polymer, and thus the layers are not stacked perfectly owing to the rounded features [32]. Additionally, long void spaces are present between adjacent polymer melt roads in the build plane. Thus, when intimate contact is maintained between the polymer melt interfaces, these interfaces exhibit a strength much lower as compared with the bulk material. These polymer interfaces act as failure initiation points, and thus give FFF parts their characteristic anisotropy.

The bond formation and strength development of the polymer melt interfaces have been described in multiple ways [33,34,35]. In this study, bond formation was described in different stages [9,36]: (1) interface heating (above glass transition temperature, T_g_), which enables the local polymer flow and polymer mobility; (2) intimate contact or close physical contact between adjacent surfaces; (3) thermal healing or diffusion at the interface; and (4) interface cooling below the glass transition temperature. In an FFF process, a polymer melt is extruded at a temperature above its glass transition temperature on to a previously deposited polymer layer that has cooled down, thus causing the interface temperature to initially rise and then rapidly fall to below the glass transition temperature [30,37]. This complex thermal history at the interface accompanied by other process conditions leads to the low inter-laminar strength at the bonded interfaces.

Previous studies have shown that applying thermal annealing alone leads to a substantial increase in the bond length at the interface, and in turn increases the thermal and mechanical properties of the parts [9,38]. The goal of the present study is to characterize and study the increase in ultimate tensile strength and develop a statistical model by subjecting the FFF parts to a post process thermal annealing, while simultaneously applying an initial static uniaxial load. The hypothesis of this work is that thermal annealing accompanied with an initial applied uniaxial load would not only assist the interface annealing, but also increase the intimate contact area, leading to a higher strength value as compared with the as built parts (parts without post processing treatments applied to them). A design of experiments (DOE) is conducted using a two-way full factorial design [39,40]. An analysis of variance (ANOVA) is performed on the data to test the significance of each factor on the strength. In addition, the changes in the mesostructure of the parts are studied to explain the changes in the difference of the average ultimate tensile strength. This study not only helps in understanding the effect of the initial applied uniaxial loads and temperature on interface healing, but also provides a technique to increase the strength of FFF parts to levels comparable to those of conventional manufacturing techniques.

## 2. Materials and Methods

Acrylonitrile butadiene (ABS) filament (Hatchbox 3D, Pomona, CA, USA) of 1.75 mm in diameter was used to print the dogbones. A new filament roll was opened from a sealed package and used immediately, but information about the filament history prior to purchase is not available. The tensile test specimen (dogbones) design is a modified version of the ASTM 638-02a standards. The specimen deviates from the ASTM standards in terms of the scaled down part dimensions to reduce the time required for part fabrication. The CAD models were designed on SOLIDWORKS 2016 (Dassault Systems, Waltham, MA, USA) and the machine language was generated using Simplify3D (Simplify3d, Cincinnati, OH, USA). The dogbones were fabricated using FFF on the Polyprinter 229 (Polyprinter; Midlothian, TX, USA) desktop printer. The nozzle diameter was 0.35 mm. The print bed and nozzle temperatures were set to 230 °C and 110 °C, respectively. The infill percentage for all the parts was kept at 100% with two perimeter shells. The given values of print settings were used because not only are they the default settings provided for the FFF machine that was used, but also they provide the best results in terms of print quality, bed adhesion, reduction in time for printing, and strength, thus reducing the chances of a print failure.

**Figure 2:**
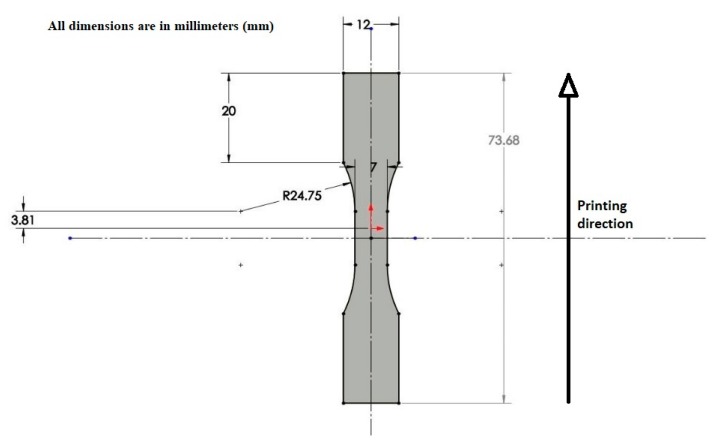
Dogbone specimen dimensions.

The dogbones were printed with their build direction parallel to its longest edge. Each part was printed individually to reduce the thermal gradient while printing each layer, and the total time to print one part was 33 min. The raster angle was kept as 0° to achieve the maximum bonded area. The part dimensions are depicted in Figure 2.

**Figure 3:**
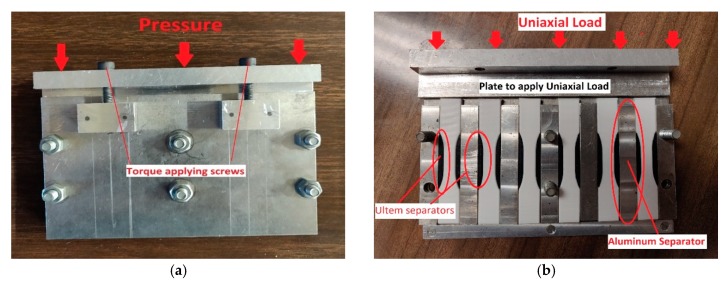
(**a**) Custom fixture to apply an initial uniaxial load. (**b**) Tensile specimen separated by aluminum separators with ULTEM parts inserted to help maintain its outer contour.

From previous work done by Hart et al., a significant improvement in fracture strength was seen for temperatures lying between 120 °C and 180 °C. On this basis, four values of temperature were chosen for thermal annealing. Three values of initial applied uniaxial loading were chosen, such that its effect on ultimate tensile strength could be studied while limiting the distortion of the parts during heat treatment. Preliminary tests on the specimens showed distortion of the part geometry owing to thermal annealing. Thus, a custom fixture was built out of aluminum with the dual purpose of preventing distortion of the part geometry and applying the initial uniaxial load along the build direction. The custom fixture is shown in Figure 3a. To prevent deformation of the curved portion of the specimens during thermal annealing, the sides of the sample were supported with parts printed using Ultem, as shown in Figure 3b. The fixture holds a total of six samples at a time. The initial uniaxial loads were applied by tightening the screws to the predefined torque level, which in turn translates into an axial force acting along the longest length of the parts. From the preliminary study, it was seen that the parts took approximately 1 h (Preheat time) to reach steady state with the oven temperature. The study by Hart et al. showed a 400% increase in fracture strength for an annealing time of 2 h at 125 °C. Further previous studies suggest that, with large annealing times, the increase in strength is accompanied by a fall in the ductility, especially for parts with low infill percentages [41]. Also, from initial tests, the parts showed low geometric distortion for an annealing time of 2 h. The oven was preheated overnight and then the fixture was placed in the preheated oven for a total of three hours, with the first hour considered as the time for the fixture to achieve thermal equilibrium with the oven temperature. Thus, the overall time for heat treatment was 3 h, which includes the time the fixture took to reach thermal equilibrium with the oven temperature. On reaching the given isothermal annealing time, the fixture was taken out of the oven and allowed to cool. Finally, the treated parts were tested together on the tensile testing machine in a randomized order. The printing and annealing of the parts took 10 days, during which the treated parts were placed in sealed bags with silica gel.

**Figure 4:**
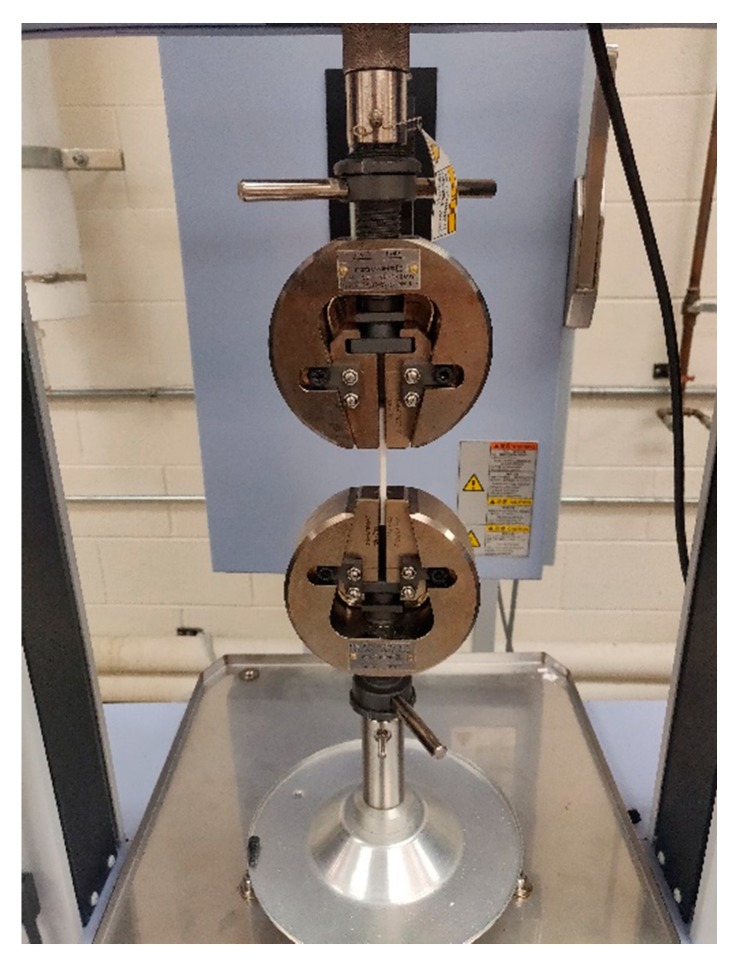
Tensile testing on the Shimadzu Universal testing machine.

The test specimens were tested on the Shimadzu Universal Testing Machine, as shown in Figure 4. A tensile load was applied on the specimens using a displacement control of 5 mm/min and a load cell of 1 kN. The raw data obtained in the form of Force and Stroke were converted to stress and strain by dividing with the cross-sectional area and the original length, respectively. The maximum stress that the part could withstand before failure was chosen as the ultimate tensile strength.

A full factorial DOE was used to conduct the experiments and analysis. From preliminary experiments, it was seen that, for temperatures beyond the glass transition temperature of ABS (i.e., 105 °C), the ultimate tensile strength increased substantially. Initial experiments showed around a 125% increase in strength for temperature values up to 180 °C. However, for the temperature of 180 °C, the parts soften, thus leading to a loss of geometric integrity. Consequently, four levels of temperature were chosen for the experiment, as given in Table 1. As no significant studies have been done on the effect of initial applied uniaxial load in the build direction at an elevated temperature, three values of loads were chosen, as stated in Table 1, such that the dimensional changes were minimized, while allowing us to study its effect on the ultimate tensile strength. The two-way full factorial design runs every combination of the levels of the two factors in Table 1. Four levels of temperature and four levels of initial applied uniaxial load were studied, which resulted in 16 treatment combinations for the full factorial design. Each treatment combination had six parts in the fixture and each treatment was replicated twice, thus the total number of parts was (16 treatments) × (6 parts/fixture) × (2 replications) = 192 parts to conduct the full factorial design. A total of 15 parts were tested for the control case. The 16 treatment combinations were performed in a randomized run order to minimize systematic biases. The parts were printed one specimen at a time on the Polyprinter 229, with each specimen requiring 33 min to print. After printing, the support brim was carefully removed, and the ends of the specimens were lightly brushed with sandpaper to ensure flatness. The oven temperature was set and adjusted with the help of a K-type thermocouple and a data acquisition system (DAQ) attached to the interior of the oven. The tolerance for the temperature was maintained at ±5 °C owing to the fluctuating nature of the oven temperature.

The parts were placed in the fixture and the bolts holding the fixture together were tightened. Also, as six parts were placed inside the fixture during each treatment, these were considered as repeat tests rather than replications because of the compounding effects present. Further, the initial uniaxial load was applied by tightening the torque screws to the desired torque value using a torque wrench. The manufacturer variation for the torque wrench accuracy is given as ±4% of the set value. After the torque was applied, the bolts holding the fixture together were further tightened to close any gaps that might have occurred between the plates parallel to the specimen faces and the separators. The fixture was then placed in the oven for a total of 3 h. After the annealing time was reached, the fixture was taken out and allowed to cool at room temperature. This procedure was repeated for each treatment combination. After the fixture was cooled, the dimensions of each specimen were taken, and the changes were recorded. After all the treatment combinations were completed, the parts were tested for tensile strength on the Shimadzu Universal testing machine. A total of 15 control specimens were used to calculate the increase in the average ultimate tensile strength of the as built parts for the studied treatment combinations from Table 1. The difference (increase or decrease) in the average ultimate tensile strength is obtained by subtracting this control value from each specimen in the different treatment combinations. It is assumed that the uncertainty observed in ultimate tensile strength follows normally distributed errors with constant variance. Microsoft Excel was used to calculate the values of stress and strain and for the plot generations. SAS (SAS Institute Inc.; Cary, NC, USA) was used to perform the statistical analysis.

## 3. Results

In this section, the DOE assumptions for the full factorial model are verified and the outcome of the ANOVA analysis is stated.

### 3.1. Raw Increase in Strength Versus Process Factors

**Figure 5:**
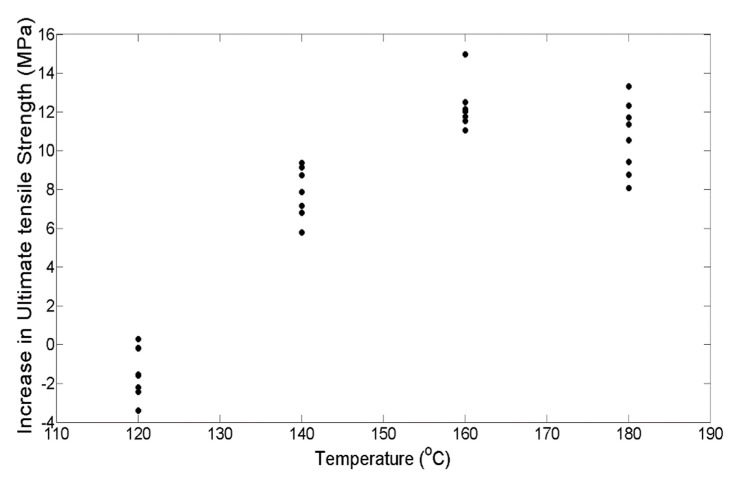
Plot showing the raw increase in strength with temperature.

The increase in tensile strength as a function of the individual factor temperature (T) is plotted in Figure 5. T shows a significant effect on the difference in the average ultimate tensile strength. The average ultimate tensile strength of the control specimens was measured to be 21.07 MPa. With an increase in temperature, the average ultimate tensile strength falls from 21.07 MPa to 20.57 MPa at 120 °C, but after that, a steady increase in the strength can be seen up to 160 °C (i.e., 34.22 MPa). This fall in strength at 120 °C could be attributed to the variability in the bond formation of the parts as well as the variability in the oven temperature. Further, as 120 °C is close to the glass transition temperature of ABS, sintering at the inter-laminar region is not significant, and thus a major improvement in the strength is not visible. From 160 °C to 180 °C, a decrease in the average strength is noticed for one set, whereas for the second replication, the average strength remains constant. It is seen that the failure mechanism shifts from brittle to slightly elastic failure at higher temperatures. The parts subjected to temperatures above 160 °C show the crazing phenomena of polymers, which causes white spots to develop prematurely along the length of the specimen, and further failure occurs at one of these white spots.

**Figure 6:**
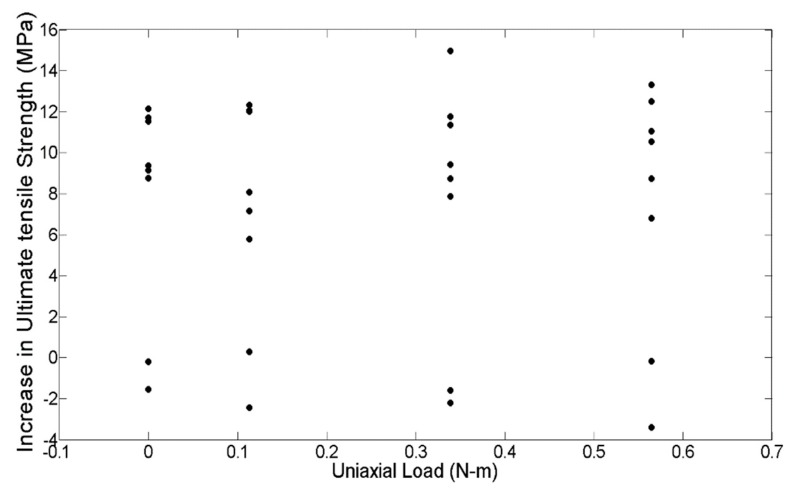
Plot showing the raw increase in strength with initial applied uniaxial load.

On the other hand, the initial applied uniaxial load (P) does not have such a notable effect on the difference in the average ultimate tensile strength, as shown in Figure 6. The maximum increase in the difference in the average strength is seen when no load is applied for all values of temperatures, except 160 °C. A significant change is not seen between the 0.11 N-m and the 0.56 N-m loads at lower temperatures, but as the temperature approaches 180 °C, the 0.56 N-m shows a larger increase in the difference of average ultimate tensile strength. Overall, however, the average ultimate strength remains somewhat constant for different values of initial applied uniaxial loadings.

**Figure 7:**
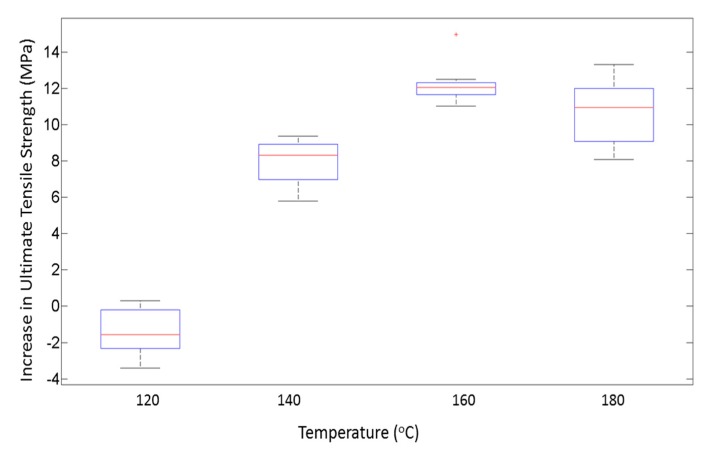
Box plot showing the variance in distribution of strength for different temperatures.

**Figure 8:**
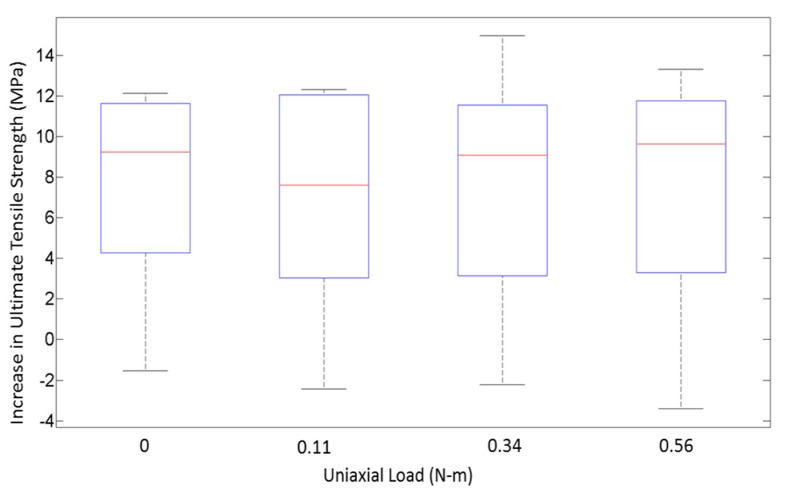
Box plot showing the variance in distribution of strength for different initial applied uniaxial loads.

**Figure 9:**
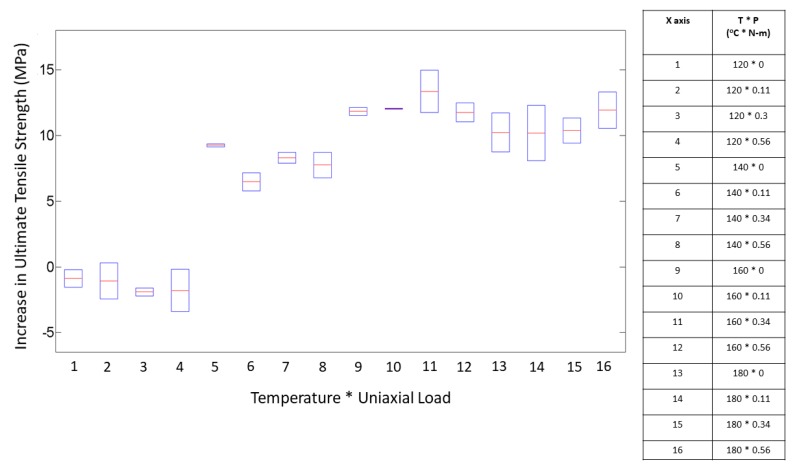
Effect of treatment levels on the ultimate tensile strength.

On comparing the variances from the box plots shown in Figure 7 and Figure 8, it is seen that the increase in ultimate tensile strength has a higher spread as a function of P as compared with T. This difference in spread is the result of the dominant effect of T at the respective P levels, as seen from Figure 5 and Figure 6. From the response versus the treatment levels plot in Figure 9, the influence of treatment levels on the difference in the average ultimate tensile strength is visible. There is a general upward trend with treatment levels owing to the dominant effect of T, whereas no trend can be seen because of the initial applied uniaxial load.

### 3.2. Two-Way Fixed Effects Model and Verification of Assumptions

For the present study, the two-way full interaction model is as follows:

(1) $$Y_{\mathrm{ijt}}=\mu \;..{+\;\alpha }_{i}{+\;\beta }_{j}+\;{\left(\alpha \beta \right)}_{\mathrm{ij}}{+\;\varepsilon }_{\mathrm{ijt}}.$$

**Figure 10:**
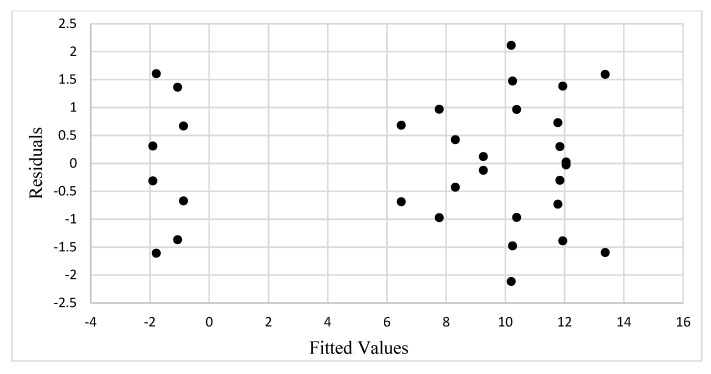
Plot showing residuals vs. fitted values.

We assume the unknown model effects are fixed and are subject to the restrictions $\sum_{i}{\left(\alpha \right)}_{i}=0$, $\sum_{j}{\left(\beta \right)}_{j}=0$, $\sum_{i}{\left(\alpha \beta \right)}_{\mathrm{ij}}=0$, and $\sum_{j}{\left(\alpha \beta \right)}_{\mathrm{ij}}=0$. In addition, as mentioned previously, the ANOVA model assumes that the error term ε_ijt_ follows a normal distribution with a constant variance and mutually independent errors. To check for constant variance, the residuals are plotted as a function of the fitted values (estimated means,$\;\hat{\;y}$), as shown in Figure 10. The estimated means are the averages of the increases in tensile strength for the two replications per treatment combination. From Figure 10, we can see that the scatter of points has no collocated residuals and is randomly distributed with somewhat equal spread throughout. Thus, the constant variance assumption seems to be satisfied.

**Figure 11:**
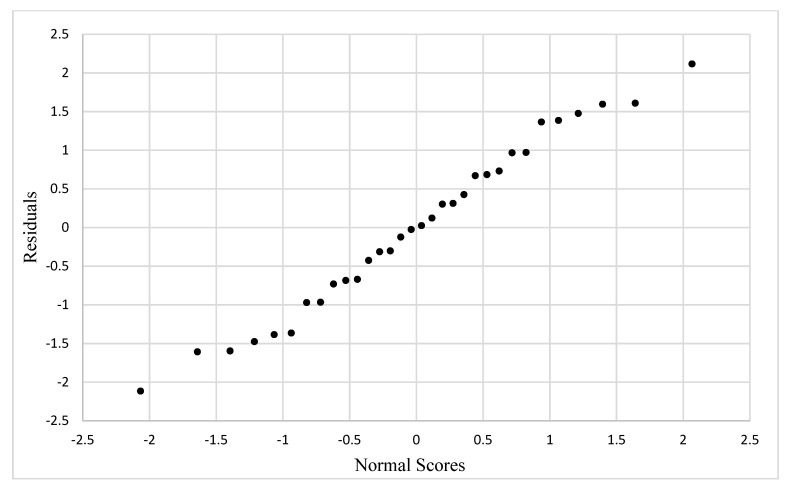
Normal probability plot for residuals.

The assumption of the normally distributed residuals can be checked by visual inspection of Figure 11, which shows the normal probability plot (NPP). The straightness of the pattern in the plot shows agreement with normality. There is a very slight “S” shape to the plot, which indicates the error distribution has tails that slightly deviate from the normal distribution, but no serious departures from normality are visible. Normality was also checked using the sample correlation coefficient $\hat{p}$ = 0.99170, which is greater than the critical value for the coefficient of correlation, c(0.1, 32) = 0.9722, thus the null hypothesis (H0) that the normality is satisfied is not rejected. As both assumptions for the model seem satisfied, a transformation is not required and we could continue with the previously chosen linear model given in Equation (1).

### 3.3. ANOVA and Factor Interactions

**Figure 12:**
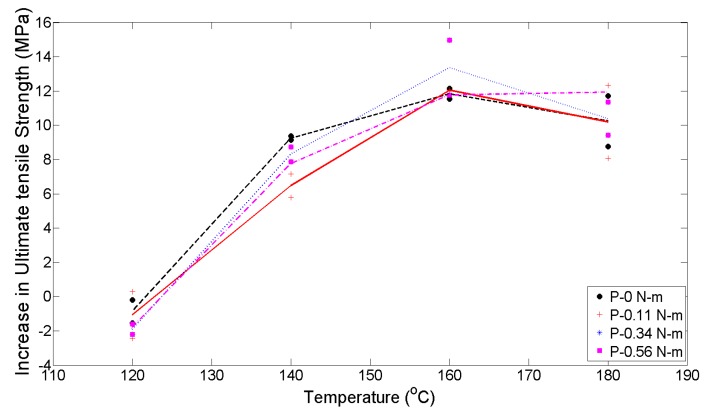
Interaction plots between temperature and initial applied uniaxial load.

From the interaction plots for the increase in ultimate tensile strength shown in Figure 12, we see that the slopes of the lines are non-zero and distinct lines are visible for each value of the initial applied uniaxial loads, thus indicating that main effects owing to both T and P, respectively, are present. The lines show similar patterns, indicating that the interaction effect between the T and P is not important. It is seen that, initially, the difference in average ultimate tensile strength of the parts decreases for the T of 120 °C, after which a steady increase in strength is seen up to 160 °C. From 160 °C to 180 °C, all values of loads lead to a fall in the difference in average ultimate tensile strength, except for the 0.56 N-m level, which stays flat. This small separation is a minor indication of a potential interaction, but it is not strong enough to support the presence of interaction effects. These results are confirmed from the ANOVA results shown in Table 2.

The ANOVA results in Table 2 show the breakdown of the variability (sum of squares) in the response, the difference in average ultimate tensile strength. The maximum contribution to this variability is due to temperature, and 96% of the variability in the response is explained by the full interaction model. It is seen that, for the interaction effects, p-value (0.7139) > 0.05 (confidence level of 95%). From this result, we can conclude that the interaction effects are negligible. As interaction effects are absent, we next check for the main effects owing to the initial applied uniaxial load and temperature. As the p-value for the initial applied uniaxial load (P) (0.8032) > 0.05 (confidence level of 95%), we conclude that the initial applied uniaxial load effects are negligible. As the interaction effects and the main effects owing to the initial applied uniaxial load are not present, the appropriate model for future work is the additive model,

(2) $$Y_{\mathrm{ijt}}=\mu ..{\;+\;\alpha }_{i}{+\;\varepsilon }_{\mathrm{ijt}},$$

with the model assumptions that $\sum_{i}{\left(\alpha \right)}_{i}=0$, where μ_∙∙_ = overall mean for all treatments, α_i_ = main effects due to temperature, and εij = error variable. For the current work, we continue with our fitted full interaction model in Equation (1).

### 3.4. Pairwise Comparisons

**Figure 13:**
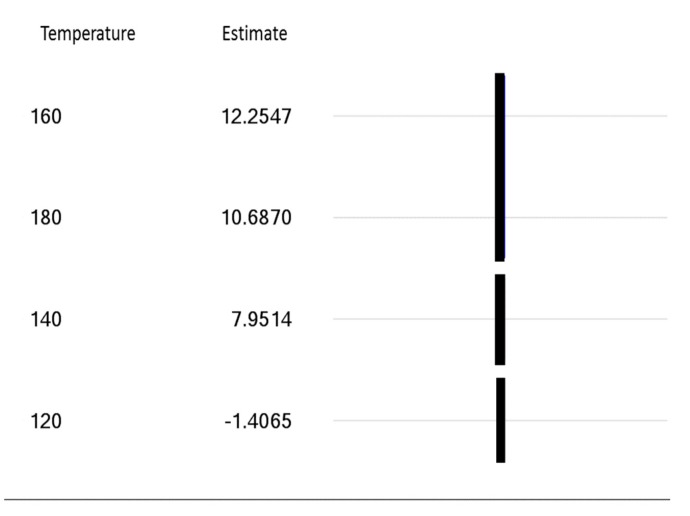
Line plot for Tukey pairwise comparison.

Figure 13 shows the Tukey pairwise comparisons result for the significant factor temperature at a 90% confidence level. This analysis of factor effects identifies how the levels of temperature are statistically distinguishable from each other. The vertical lines connect the temperature levels that are statistically the same. In this case, the two lowest temperatures have mean differences in the average ultimate tensile strength that are statistically distinguishable from each other and from the two higher temperatures. This implies that progressively higher increases in ultimate tensile strength can be achieved as the temperature rises from 120 °C to 140 °C to 160 °C. However, increasing the temperature to 180 °C would not yield an additional benefit in ultimate tensile strength.

## 4. Discussion

From the ANOVA, it is seen that, for a confidence level of 95%, the main effects owing to initially applied uniaxial loading and the interaction effects between temperature and initial applied uniaxial loading are absent. For 120 °C, which is close to the glass transition temperature, an initial decrease in the difference of the average ultimate tensile strength is seen. However, further increasing the temperature to 160 °C shows a somewhat linear increase in the difference in average ultimate tensile strength. An additional increase in the annealing temperature to 180 °C shows a fall in the difference in the average tensile strength. A study of the cross-sectional images captured with the help of a camera attached to an optical microscope helps shed some light on the overall behavior of the specimen undergoing thermal annealing.

**Figure 14:**
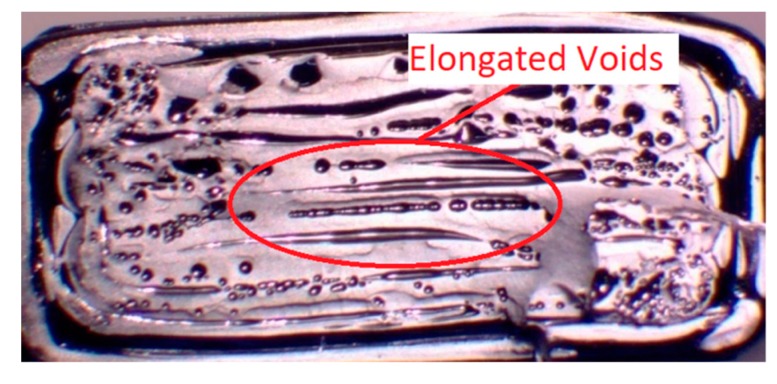
Cross section of failure for an as built part.

**Figure 15:**
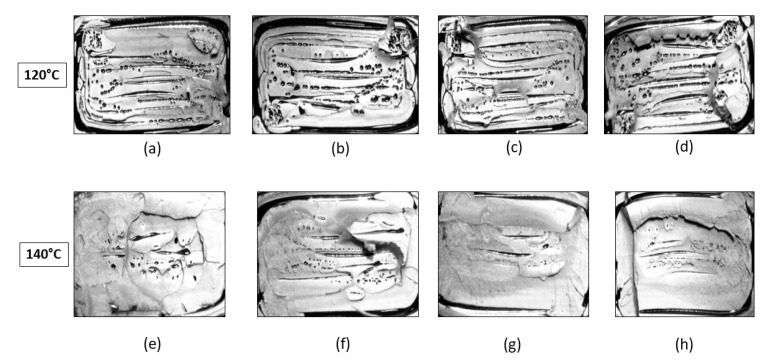
Cross section of failure for parts treated to (**a**) 120 °C and 0 lb.-in, (**b**) 120 °C and 1 lb.-in, (**c**) 120 °C and 3 lb.-in, (**d**) 120 °C and 4 lb.-in, (**e**) 140 °C and 0 lb.-in, (**f**) 140 °C and 1 lb.-in, (**g**) 140 °C and 3 lb.-in, and (**h**) 140 °C and 5 lb.-in.

**Figure 16:**
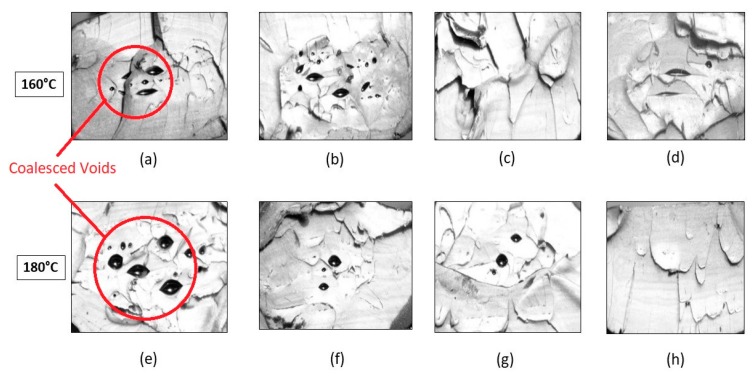
Cross section of failure for parts treated to (**a**) 160 °C and 0 lb.-in, (**b**) 160 °C and 1 lb.-in, (**c**) 160 °C and 3 lb.-in, (**d**) 160 °C and 4 lb.-in, (**e**) 180 °C and 0 lb.-in, (**f**) 180 °C and 1 lb.-in, (**g**) 180 °C and 3 lb.-in, and (**h**) 180 °C and 5 lb.-in.

Figure 14 shows the cross section of failure for an as built part. The presence of long voids along the rasters and insufficient bonding at the polymer melt interfaces can be seen. Figure 15 and Figure 16 show the cross sections where failure occurred for the post processed parts. On visual inspection of the cross sections, no significant effects due to initially applied uniaxial loading are seen on the mesostructure or void pattern, thus reaffirming the results obtained through ANOVA that the effects due to initially applied uniaxial load may be ignored for a similar fixture. This, however, does not imply that a load along the build direction does not help in increasing the contact area, and thus improving the bonding, rather it is seen that, as the fixture applies only an initial static load, it fails to continuously do so when the specimens undergo annealing. As annealing progresses, the specimen contracts and the custom fixture is incapable of maintaining the initially applied uniaxial loading in the build direction, thus showing no effect on strength owing to the load. Conversely, the effect of temperature is visible in the cross-sectional images taken along both the axis parallel to the length as well as the axis parallel to the laminas (x–y plane).

**Figure 17:**
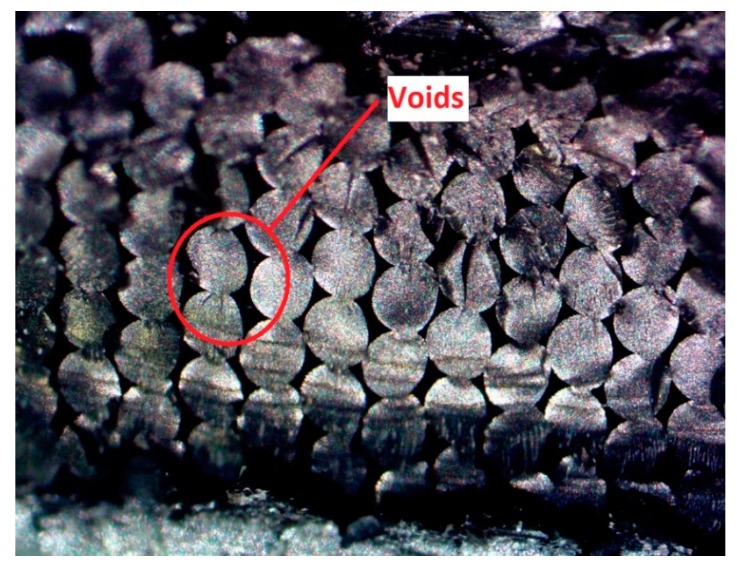
Voids and bond length at the polymer interface for as built part.

**Figure 18:**
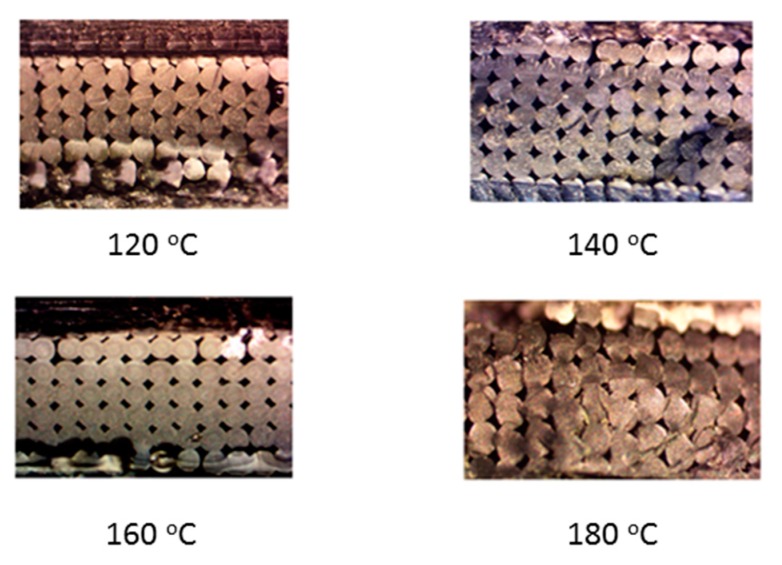
Changes in bond length for different annealing temperatures.

Visual inspection of the untreated part in Figure 14 indicates the presence of elongated voids along the raster directions owing to the rounded nature of the extruded filaments. Also, multiple circular voids are visible owing to the improper bonding occurring between the inter-laminar regions. The presence of these voids reduces the total bonded area at the polymer–polymer interface, thus reducing the total bonded area and the ultimate tensile strength. As seen from Figure 15, the cross sections of the parts annealed to 120 °C do not show any major changes in the number of voids or their sizes. This is consistent with the results obtained from the tensile testing and ANOVA analysis, which show that the increase in the difference in the average ultimate tensile strength at 120 °C is insignificant, rather a slight decrease in this difference is noticed. But as the temperature increases beyond 120 °C void coalescence takes place as shown in Figure 16. To obtain the images exposing the bond length, initially, a notch was cut into the specimen and then it was placed in liquid nitrogen for five minutes to make it brittle. The frozen specimen was then clamped in a table vise and struck with a hammer, resulting in the cross section along the length being visible. This cross section was then observed under an optical microscope to study the changes in bond length. As seen from Figure 17 and Figure 18, the bond length at the polymer interface is not affected to a great extent for 120 °C and the failure during testing occurs as a result of laminar separation. At 140 °C, the elongated voids present between adjacent filaments almost disappear. The total bond length also increases, resulting in the increase of ultimate tensile strength, but failure still occurs at the laminar interface. Further increase to 160 °C causes the elongated voids to completely disappear, and instead larger circular voids are formed owing to the migration and coalescence of the smaller voids [9]. Figure 18 also shows that the bond length increases significantly, causing the parts to fail at a much higher value of ultimate tensile strength. The zone of failure for parts annealed at 160 °C is where most voids are present owing to coalescence and not at the polymer–polymer interface because the greater bonding is achieved by annealing. Also, crazing or whitening of the specimen cross section occurs before the actual failure of the part at this location. The final temperature of 180 °C shows an increase in the size of the voids and bond-length, but these changes in the mesostructure are not drastically different than those seen at 160 °C. This observation is in line with the Tukey pairwise comparison, which shows that the mean differences in the average ultimate tensile strength for parts annealed at 160 °C and 180 °C are not statistically distinguishable. The increase in the average ultimate tensile strength for the various temperature values is shown in Table 3.

**Figure 19:**
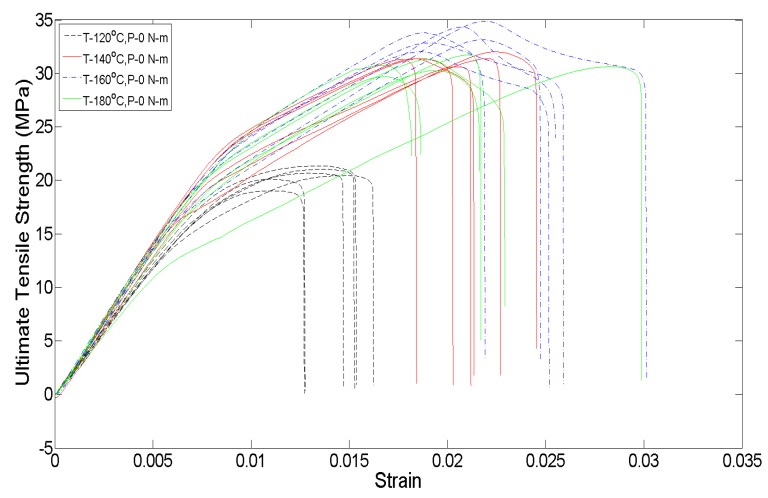
Engineering stress–strain plot for different annealing temperatures.

Also, apart from the changes in the mesostructure of the specimen, which can be associated with the variations in the average ultimate tensile strength, a change in the ductility of the parts is seen in the engineering stress–strain. Figure 19 shows the engineering stress–strain plots for the no initially applied uniaxial load condition for the first replication of parts (each treatment has six repeat tests). As the annealing temperature increases from 120 °C to 160 °C, the part behavior shifts from brittle failure to a more elastic-brittle failure, with some yielding occurring for parts at 160 °C. The ductility of the parts falls for 180 °C as compared with 160 °C, and this is seen to be because of the presence of larger voids at 180 °C. When yielding for the parts at 160 °C, the crazing phenomena is noticed at multiple regions along the length of the part before part failure, but for 180 °C, after crazing occurs, the part failure immediately follows with some or no yielding.

**Figure 20:**
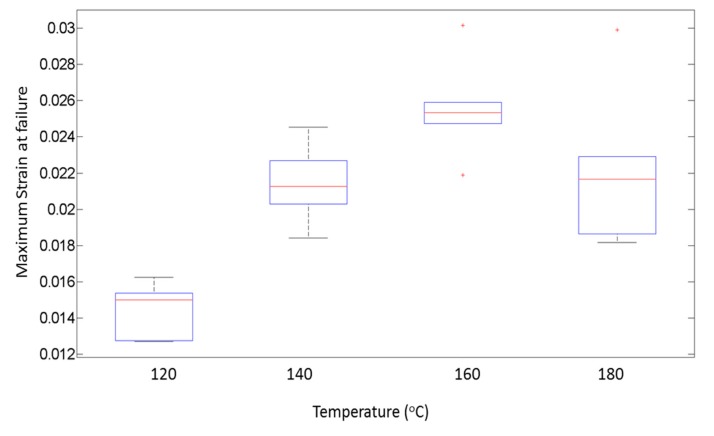
Maximum strain at failure for corresponding temperatures.

Figure 20 shows the maximum strain achieved in a part before failure for the corresponding values of temperature. It is seen that, as temperature is increased from 120 °C to 160 °C, the average value of maximum strain shows an upward trend. This upward trend can be associated with the increase in ductility of the parts. For 180 °C, the average maximum strain decreases as compared with 160 °C, but it is also seen that the variability in the maximum strain is substantially higher than other values of temperature. At 180 °C, the voids coalesce and are larger in size than at 160 °C, causing the failure to occur at a reduced value of stress and strain.

## 5. Conclusions

In this study, a design of experiments approach was used to characterize the increase in tensile strength owing to the effect of thermal annealing with the simultaneous application of an initial uniaxial load. A fixture was built and the dogbone specimens were placed in it to undergo thermal annealing in an oven while initially applying uniaxial loads. Four levels of temperature and loads were chosen (16 treatment combinations) for the two-way full factorial design of experiments. The post processed specimens were tested on a universal testing machine and the difference in the average ultimate tensile test between the treated and untreated parts was calculated. The analysis of variance (ANOVA) revealed the lack of interaction effects between the temperature and initially applied uniaxial load and the absence of main effects due to initially applied uniaxial loading alone at a 90% significance level for the fixture used. Thus, for future models that use a similar fixture, only the main effects of the temperature should be considered, whereas the interaction effects and main effects due to initially applied uniaxial loading can be ignored. A study of the cross sections of the tested parts shows no significant effects due to the initially applied uniaxial loading on the mesostructure, and thus is in good agreement with the ANOVA results. It is found that temperature main effects have the largest contribution to the increase in tensile strength. The tensile strength shows an almost linear increase from 120 °C to 160 °C and a slight decrease in strength at 180 °C. Also, for the temperature level of 160 °C, the maximum value for the increase in tensile strength was obtained with an increase of 89% (34.22 MPa) and the strength of these parts approaches close to the ultimate tensile strength of ABS, that is, 40 MPA. From the work done in this paper, it is expected to achieve theoretical insights into the increase in ultimate tensile strength due to thermal annealing and initially applied uniaxial loading, and thus will facilitate the use of additively manufactured parts with greatly reduced anisotropy and strengths comparable to those of injection molded parts. Also, it would facilitate future studies of fracture failure in thin beam as well as solid beam structures for FFF parts.

## Figures and Tables

**Table 1:** Temperature and initial applied uniaxial load levels for the design of experiments.

Levels	Temperature (°C)	Initial Applied Uniaxial Load (lb.-in)	Initial Applied Uniaxial Load (N-m)
1	120	0.0	0.00
2	140	1.0	0.11
3	160	3.0	0.34
4	180	5.0	0.56

**Table 2:** Analysis of variance table.

Source	Degrees of Freedom	Type III Sums of Squares	Mean Square	F Value	Pr > F
Temperature (T)	3	897.82	299.27	124.16	<0.0001
Initial Applied Uniaxial Load (P)	3	2.39	0.797	0.33	0.8032
T × P	9	14.822	1.646	0.68	0.7139
Error	16	38.565	2.41		
Corrected Total	31	953.607			

**Table 3:** Percentage increase in strength for different annealing temperatures.

Levels	Temperature (°C)	Percentage Increase in the Average Ultimate Tensile Strength
1	120	−6
2	140	70
3	160	89
4	180	82
